# Differences between COVID-19-vaccinated and unvaccinated participants from Croatia

**DOI:** 10.3325/cmj.2022.63.508

**Published:** 2022-12

**Authors:** Ljiljana Kaliterna Lipovčan, Zvjezdana Prizmić-Larsen, Renata Franc

**Affiliations:** 1 Ivo Pilar Institute of Social Sciences, Zagreb, Croatia; 2 Department of Psychological & Brain Sciences, Washington University in St. Louis, St. Louis, MO

## Abstract

**Aim:**

To compare Croatian participants vaccinated against coronavirus disease 2019 (COVID-19) and unvaccinated participants in terms of socio-demographic, personal, social, and COVID-19-related variables.

**Methods:**

From August till December 2021, 721 (465 vaccinated and 256 unvaccinated) participants completed an online survey about socio-demographic (age, sex income, education, marital status), personal (well-being indicators, personality measures and health), social (trust in experts, trust in government), and COVID-19-related characteristics (fear of COVID-19, history of COVID-19 infection). Differences between the groups were assessed with discriminant analysis.

**Results:**

The variables that best discriminated between vaccinated and unvaccinated participants were higher trust in experts, no history of COVID-19 infection, older age, higher fear of COVID-19, and intellect.

**Conclusion:**

The study points to the importance of trust in experts in the promotion of COVID-19 vaccine.

Vaccination against coronavirus disease 2019 (COVID-19) remains the most important measure for controlling the pandemic (1,2). Vaccines have also played an important role in the prevention of other diseases, and vaccination of children against certain diseases is compulsory in most of the developed countries (3). Despite all of this, some people remain vaccine hesitant. In 2019, the World Health Organization (WHO) listed vaccine hesitancy (4) as one of the ten threats to global health (5). The percentage of people worldwide who have been vaccinated against COVID-19 is still not at 70% of total population, the targeted coverage by mid-2022 (6). As of March 24, 2022, a total of 57% of people were fully vaccinated globally, with the figures in Europe and Croatia being 62% and 55%, respectively (7).

To help public health officials address vaccine hesitancy it is important to understand the factors that influence it (2,8,9). Factors associated with COVID-19-vaccine hesitancy were similar to those related to hesitancy to other vaccines, and were grouped as those related to vaccine attributes, political factors, and individual characteristics (9). Among them the most studied were various socio-demographic characteristics. Vaccine hesitancy was associated with younger age (2,10,11), female sex (2,11), lower education (2,10,11), lower income (2), and non-liberal political views (10). Little attention has been paid to the role of personality in the intention to get vaccinated (12). However, many studies explored its role in precautionary behaviors. For example, agreeableness and conscientiousness were related to complying with guidelines in US (13) and Croatia (14), while extraversion was negatively related to adherence to social distancing (15). People who experience higher fear of COVID-19 (16-20) and people with a higher perceived risk from COVID-19 infection were more willing to get vaccinated (11). Other factors associated with willingness to receive COVID-19 vaccine were trust in different institutions, and attitudes and beliefs toward vaccine and its benefits (21). A study across 12 national samples (21) found the strongest correlates of vaccine acceptance to be the level of worry about COVID-19 and trust in experts.

Many studies have examined hesitancy/willingness to get the vaccine against COVID-19 (2,10,11,21), however, to our knowledge no studies specifically explored differences between vaccinated and unvaccinated people. This study was conducted at a later stage of the pandemic when vaccines in Croatia were widely available, and when their efficacy was well known. Thus, our aim was to assess the differences in socio-demographic, personal, social, and COVID-19-related variables according to vaccination status. We hypothesized that vaccinated people would differ most from unvaccinated people in age, fear of COVID-19, trust in experts, and the history of COVID-19 infection (11,18,20,22).

## Participants and methods

### Participants

From August till December 2021, 1136 participants completed the online survey. The study included 721 participants (465 vaccinated and 256 unvaccinated) who answered the questions related to COVID-19. Socio-demographic characteristics of the sample: age, gender, education level, marital status, and income, are presented in Table 1.

**Table 1:** Socio-demographic characteristics and descriptive statistics for the total sample (N = 721), vaccinated participants (N = 465), and unvaccinated participants (N = 256)

	Total sample	Vaccinated	Unvaccinated
	No. (%)		
Gender			
female	583 (81)	375 (81)	208 (81)
male	138 (19)	90 (19)	48 (19)
Education level*			
high school^†^	164 (23)	95 (20)	69 (27)
bachelor’s degree	411 (57)	262 (57)	149 (58)
post-graduate	144 (20)	106 (23)	38 (15)
Marital status			
married or in relationship	571 (79)	363 (78)	208 (81)
other	150 (79)	102 (22)	48 (19)
Income* (in Euro^‡^)			
<267	37 (5)	19 (4)	18 (8)
268-667	237 (35)	155 (35)	82 (35)
668-1600	331 (49)	223 (50)	108 (47)
>1601	72 (11)	48 (11)	24 (10)
History of COVID-19 infection			
yes	174 (24)	77 (17)	97 (38)
no	547 (76)	388 (83)	159 (62)
	mean (standard deviation)		
Age	43.0 (12.34)	44.6(12.57)	40.1 (11.35)
Fear of COVID-19 scale	1.7 (0.68)	1. 8 (0.69)	1.6 (0.67)
Health	4.0 (0.88)	3.9 (0.87)	4.0 (0.88)
Extraversion	3.5 (0.90)	3.5 (0.90)	3.5 (0.89)
Neuroticism	2.8 (0.94)	2.8 (0.92)	2.9 (0.97)
Conscientiousness	3.6 (0.86)	3.6 (0.86)	3.6 (0.84)
Agreeableness	4.3 (0.63)	4.3 (0.64)	4.3 (0.64)
Intellect	4.0 (0.66)	4.0 (0.65)	3.9 (0.67)
Internal locus of control	5.2 (0.98)	5.2 (0.96)	5.2 (1.02)
External locus of control	9.5 (3.39)	9.3 (3.44)	9.9 (3.27)
Life satisfaction	7.0 (2.13)	7.1 (2.03)	6.9 (2.29)
Positive affect	5.0 (1.11)	5.0 (1.12)	5.0 (1.12)
Negative affect	3.3 (1.10)	3.3 (1.13)	3.4 (1.04)
Trust in experts	6.8 (2.31)	7.4 (1.88)	5.6 (2.60)
Trust in government	2.8 (2.33)	3.6 (2.15)	2.4 (2.10)

*Total N is lower due to missing data.

†Every participant completed elementary school.

‡Average monthly income per person; 7.5 HRK = 1 EUR on November 30, 2021.

### Methods

The data were collected as a part of the fourth wave of the Croatian Longitudinal Study on Well-being (CRO-WELL). The survey started in 2017 when the first sample of participants (N = 5080) was recruited to participate in an online survey on well-being. The survey was advertised in the media, online forums, social networks, and web sites. Everyone aged 18+ who was interested in joining the survey was able to access it using the link provided at the research website. At the start of the survey, participants were informed that the participation was voluntary and that the data would be used only for scientific purposes. Anonymity was assured by a system of tokens given to every participant before starting the survey. The second and third survey were conducted in the way that two research waves were one year apart, while the fourth and the third wave were two years apart. The second wave of the survey included 2752, the third 1891, and the fourth 1136 participants. The study was approved by the Ethics Committee of Ivo Pilar Institute of Social Sciences (11-73/14-2061).

The research was conducted via an online application, which comprised a comprehensive battery of questionnaires on well-being, life events, socio-demographic characteristics, social and personal variables, and COVID-19.

### Instruments

*Personal variables* included well-being, personality measures, and health status. Life satisfaction was assessed with the question “All things considered, how satisfied are you with your life as a whole nowadays?” and rated from 0 (extremely dissatisfied) to 10 (extremely satisfied). The Scale of Positive and Negative Experience (23) measured positive affect (positive, good, pleasant, happy, joyful, contented) and negative affect (negative, bad, unpleasant, sad, afraid, angry). To assess how often they felt a particular affect during the last month, participants rated each item from 1 (never) to 7 (always). Both scales showed good reliability measured by Cronbach’s alpha coefficient (α): α = 0.94 for Positive Affect and α = 0.89 for Negative Affect. Big-Five personality traits (extraversion, neuroticism, conscientiousness, agreeableness, intellect) were assessed with a 15-item version of the International Personality Item Pool (24). Participants rated how accurately each item described them from 1 (extremely inaccurately) to 5 (extremely accurately). The scales showed good reliability: α = 0.78 for Extraversion, α = 0.80 for Neuroticism, α = 0.72 for Conscientiousness, α = 0.75 for Agreeableness, and α = 0.65 for Intellect. The brief 9-item Locus of Control Scale (25) measured the degree of belief that life outcomes were controlled by one’s own actions, as a measure of internal locus of control, or by chance or powerful others, as a measure of external locus of control. The ratings ranged from 1 (strongly disagree) to 7 (strongly agree). Both scales showed good reliability: α = 0.78 for Internal and α = 0.82 for External Locus of Control. Health status was assessed with the question “In general how would you describe your health?” and was rated from 1 (poor) to 5 (excellent).

*Social variables*. The participants assessed how much they trusted each of the 10 listed institutions when it came to dealing with the pandemic, from 0 (not at all) to 10 (completely). Two scales were defined: Trust in Government (government, parliament, politicians, local and national authorities in disaster situations, local government) and Trust in Experts (physicians and medical stuff, scientists, educational institutions). Both scales showed good reliability: α = 0.87 for Trust in Experts and α = 0.94 for Trust in Government.

*COVID-19-related variables*. Fear of COVID-19 scale (26,27) assessed fear of COVID-19 with 7 items rated from 1 (strongly disagree) to 5 (strongly agree). The scale showed the reliability of α = 0.83. The vaccination status was assessed with the question “Did you receive a COVID-19 vaccine (at least one dose)?”, while the history of COVID-19 infection was assessed with the question: “Have you been infected with COVID-19?”, both answered with “yes” or “no”.

Instruments originally in the English language were translated into Croatian by two independent researchers, and back-translated into English by an expert in both languages. The final wording of items in the Croatian language was agreed upon by all three of them.

### Statistical analysis

Data are summarized using descriptive statistics. Categorical variables are presented as counts (percentage) and continuous variables as means (standard deviation). Pearson correlation analyses were performed. Discriminant function analysis was conducted to determine the variables/indicators that best discriminated between the vaccinated and unvaccinated group. Indicators included socio-demographic (age, gender, income, education, marital status), personal (well-being, personality, health), social (trust in institutions), and COVID-19 related variables (fear of COVID-19; COVID-19 infection). Statistical analysis was performed with IBM SPSS Statistics for Windows, version 27.0 (IBM Corp., Armonk, NY, USA).

## Results

Descriptive statistics by vaccination status are presented in Table 1, and intercorrelations between the variables are presented in Table 2. Participants without COVID-19 infection tended to be vaccinated, which was the strongest association between COVID-19 infection status and other variables. Being vaccinated was positively associated with fear of COVID-19, intellect, trust in experts, and trust in government, while it was negatively associated with external locus of control.

**Table 2:** Intercorrelations between variables for the total sample (N = 721)

	2	3	4	5	6	7	8	9	10	11	12	13.	14.	15.	16.
1. Fear of COVID-19 scale	-0.29	-0.09	0.21	0.07	0.02	-0.06	-0.22	0.22	-0.19	-0.20	0.23	0.03	0.05	0.02	0.11
2. Health	-	0.09	-0.20	0.06	0.01	0.05	0.35	-0.31	0.35	0.43	-0.32	0.13	0.09	0.01	-0.07
3. Extraversion	-	-	-0.27	0.13	0.22	0.24	0.16	-0.20	0.20	0.24	-0.21	-0.01	0.03	-0.02	0.02
4. Neuroticism	-	-	-	-0.08	-0.10	-0.17	-0.24	0.29	-0.31	-0.34	0.38	-0.11	-0.14	0.01	-0.05
5. Conscientiousness	-	-	-	-	0.20	0.04	0.05	-0.05	0.11	0.09	-0.07	-0.01	-0.01	-0.02	-0.03
6. Agreeableness	-	-	-	-	-	0.21	0.07	-0.04	0.04	0.09	0.03	0.03	0.02	-0.04	-0.04
7. Intellect	-	-	-	-	-	-	0.13	-0.12	0.08	0.12	-0.07	0.10	-0.01	0.02	0.11
8. Internal locus of control	-	-	-	-	-	-	-	-0.53	0.52	0.58	-0.43	0.20	0.15	-0.01	0.03
9. External locus of control	-	-	-	-	-	-	-	-	-0.48	-0.46	0.37	-0.22	-0.16	0.01	-0.08
10. Life satisfaction	-	-	-	-	-	-	-	-	-	0.72	-0.55	0.21	0.20	-0.07	0.05
11. Positive affect	-	-	-	-	-	-	-	-	-	-	-0.72	0.19	0.19	-0.06	0.01
12. Negative affect	-	-	-	-	-	-	-	-	-	-	-	-0.14	-0.20	0.09	-0.05
13. Trust in experts	-	-	-	-	-	-	-	-	-	-	-	-	0.59	0.03	0.36
14. Trust in government	-	-	-	-	-	-	-	-	-	-	-	-	-	0.03	0.25
15. History of COVID-19 infection (0 = yes, 1 = no)	-	-	-	-	-	-	-	-	-	-	-	-	-	-	0.24
16. Vaccination status (0 = no, 1 = yes)	-	-	-	-	-	-	-	-	-	-	-	-	-	-	-

In discriminant function analysis, tests of the equality of group means showed that six indicators (trust in experts, trust in government, history of COVID-19 infection, age, fear of COVID-19, and intellect) significantly (*P* < 0.001) contributed to the differences between the groups (Table 3). The analysis resulted in one significant discriminant function with the overall model, Wilks’s λ = 0.76 (*χ^2^* (20, N = 675) = 185.50, *P* < 0.001; *R_C_*  _=_  0.49) accounting for 24% of between-group variability. The discriminant function had the strongest relationship with trust in experts, followed by trust in government, history of COVID-19 infection, age, fear of COVID-19, and intellect. A closer analysis of the standardized coefficient showed that trust in government had a low weight, which indicated that it did not contribute to differentiation between the groups. The variables that best differentiated vaccinated from unvaccinated participants were higher trust in experts, no history of COVID-19 infection, and older age. Higher fear of COVID-19 and intellect, although less successful as discriminating variables, still contributed to differentiation between the groups. The group centroid for the vaccinated group was 0.41, and that for the unvaccinated group was -0.78. The cross-validated classification showed that 75% of the sample was correctly classified into two groups.

**Table 3:** The summary of discriminant function analysis between vaccinated and unvaccinated groups

	Structure matrix	Standardized coefficients
Trust in experts	0.68	0.71
History of COVID-19 infection (0 = yes, 1 = no)	0.44	0 .50
Trust in government institutions	0.43	0.05
Age	0 .32	0.30
Fear of COVID-19 scale	0.21	0.21
Intellect	0.20	0.20
Education	0.16	0.06
External locus of control	-0.13	-0.08
Negative affect	-0.12	-0.22
Life satisfaction	0.10	0.15
Health	-0.10	-0.20
Agreeableness	-0.10	-0.11
Income	0.09	0.10
Marital status (0 = other, 1 = married or in a relationship)	-0.09	-0.09
Neuroticism	-0.09	0.01
Internal locus of control	0.07	-0.04
Gender (0 = female, 1 = male)	0.06	-0.01
Conscientiousness	-0.04	-0.06
Positive affect	0.04	-0.25
Extraversion	0.03	0.02

## Discussion

This study identified differences in a range of socio-demographic, personal, and social characteristics between vaccinated and unvaccinated participants at a later stage of the COVID-19 pandemic (autumn 2021) in Croatia. We confirmed our hypothesis that the main characteristics of vaccinated participants were higher trust in experts, no history of COVID-19 infection, older age, and greater fear of COVID-19. Another indicator that significantly discriminated between the groups was intellect, a personality trait linked to intelligence and tendency to embrace new experiences (28). It is unsurprising that this personality trait played a role at an uncertain time when people needed to embrace newly developed vaccines.

In other studies, vaccine hesitancy was associated with lack of trust in government and health care institutions and with external health locus of control (10,29). Individuals not having been infected with COVID-19 (18) and those who expressed fear of COVID-19 were more likely to receive the vaccine (20). To understand the role of fear and the ways how people respond to COVID-19, scientists proposed the terror management health model (20,30). According to this model, the thoughts of death can increase the motivation for healthy behavior, such as vaccination, in order to reduce the feeling of threat and helplessness.

In our study, no socio-demographic characteristic, except age, significantly differentiated between vaccinated and unvaccinated participants, although in other studies vaccination hesitancy was associated with being younger, female, less educated (1,2,10,11), and healthier (31). Well-being variables also did not differentiate between the groups. However, in a UK survey, vaccination increased psychological well-being by decreasing the perceived likelihood of contracting COVID-19 and increasing engagement in social activities (32). Also, in a survey conducted in 35 countries, happier people complied more with anti-pandemic measures (33). These factors might have played an important role in vaccination intention at the beginning of the pandemic, when vaccine was not yet widely available. Our study was performed at a later stage, when vaccine was available for all citizens, so that personal and social characteristics became more important than well-being measures and socio-demographics.

Our study has some limitations. Its cross-sectional and correlational design limits inferences about causality. We used a convenience sample, meaning that the results cannot be generalized to the entire population. All measures were self-reports and are potentially subject to measurement biases, so we suggest that future studies include objective measures.

To conclude, our study showed that the most prominent characteristic that differed between vaccinated and unvaccinated participants was trust in experts (physicians, scientists, and educational institutions). People who were vaccinated trusted experts more than those who were vaccine-hesitant. This study clearly shows that the key for successful fight against the pandemic in Croatia are trustworthy and reliable actors in promoting vaccination against COVID-19.
